# GS-CA Compounds: First-In-Class HIV-1 Capsid Inhibitors Covering Multiple Grounds

**DOI:** 10.3389/fmicb.2019.01227

**Published:** 2019-06-20

**Authors:** Kamal Singh, Fabio Gallazzi, Kyle J. Hill, Donald H. Burke, Margaret J. Lange, Thomas P. Quinn, Ujjwal Neogi, Anders Sönnerborg

**Affiliations:** ^1^ Department of Molecular Microbiology and Immunology, University of Missouri, Columbia, MO, United States; ^2^ Bond Life Sciences Center, University of Missouri, Columbia, MO, United States; ^3^ Division of Clinical Microbiology, Department of Laboratory Medicine, Karolinska Institute, Stockholm, Sweden; ^4^ Department of Chemistry, University of Missouri, Columbia, MO, United States; ^5^ Department of Biochemistry, University of Missouri, Columbia, MO, United States; ^6^ Division of Infectious Diseases, Department of Medicine Huddinge, Karolinska Institute, Stockholm, Sweden

**Keywords:** human immunodeficiency virus, capsid, assembly, small molecules, inhibitors, disassembly, uncoating

## Abstract

Recently reported HIV-1 capsid (CA) inhibitors GS-CA1 and GS-6207 (an analog of GS-CA1) are first-in-class compounds with long-acting potential. Reportedly, both compounds have greater potency than currently approved anti-HIV drugs. Due to the limited access to experimental data and the compounds themselves, a detailed mechanism of their inhibition is yet to be delineated. Using crystal structures of capsid-hexamers bound to well-studied capsid inhibitor PF74 and molecular modeling, we predict that GS-CA compounds bind in the pocket that is shared by previously reported CA inhibitors and host factors. Additionally, comparative modeling suggests that GS-CA compounds have unique structural features contributing to interactions with capsid. To test their proposed binding mode, we also report the design of a cyclic peptide combining structural units from GS-CA compounds, host factors, and previously reported capsid inhibitors. This peptide (Pep-1) binds CA-hexamer with a docking score comparable to GS-CA compounds. Affinity determination by MicroScale thermophoresis (MST) assays showed that CA binds Pep-1 with a ~7-fold better affinity than well-studied capsid inhibitor PF74, suggesting that it can be developed as a possible CA inhibitor.

## Introduction

Exceptional developments in combination antiretroviral therapy (cART) have transformed HIV/AIDS from a deadly pandemic to a chronic and manageable disease (Antiretroviral Therapy Cohort, 2017). If administered efficiently, cART significantly reduces morbidity and mortality of HIV-infected individuals, both in resource-rich and in low- and middle-income countries (Quinn, 2008; Sabin, 2013; May et al., 2014; Harries et al., 2016; Teeraananchai et al., 2017). However, emerging drug resistance mutations (DRMs) and the side effects of approved anti-HIV drugs continue to threaten the desired outcome of cART. Hence, current efforts are focused on the discovery of new antivirals acting through novel mechanisms and/or directed to new targets.

**Figure 1:**
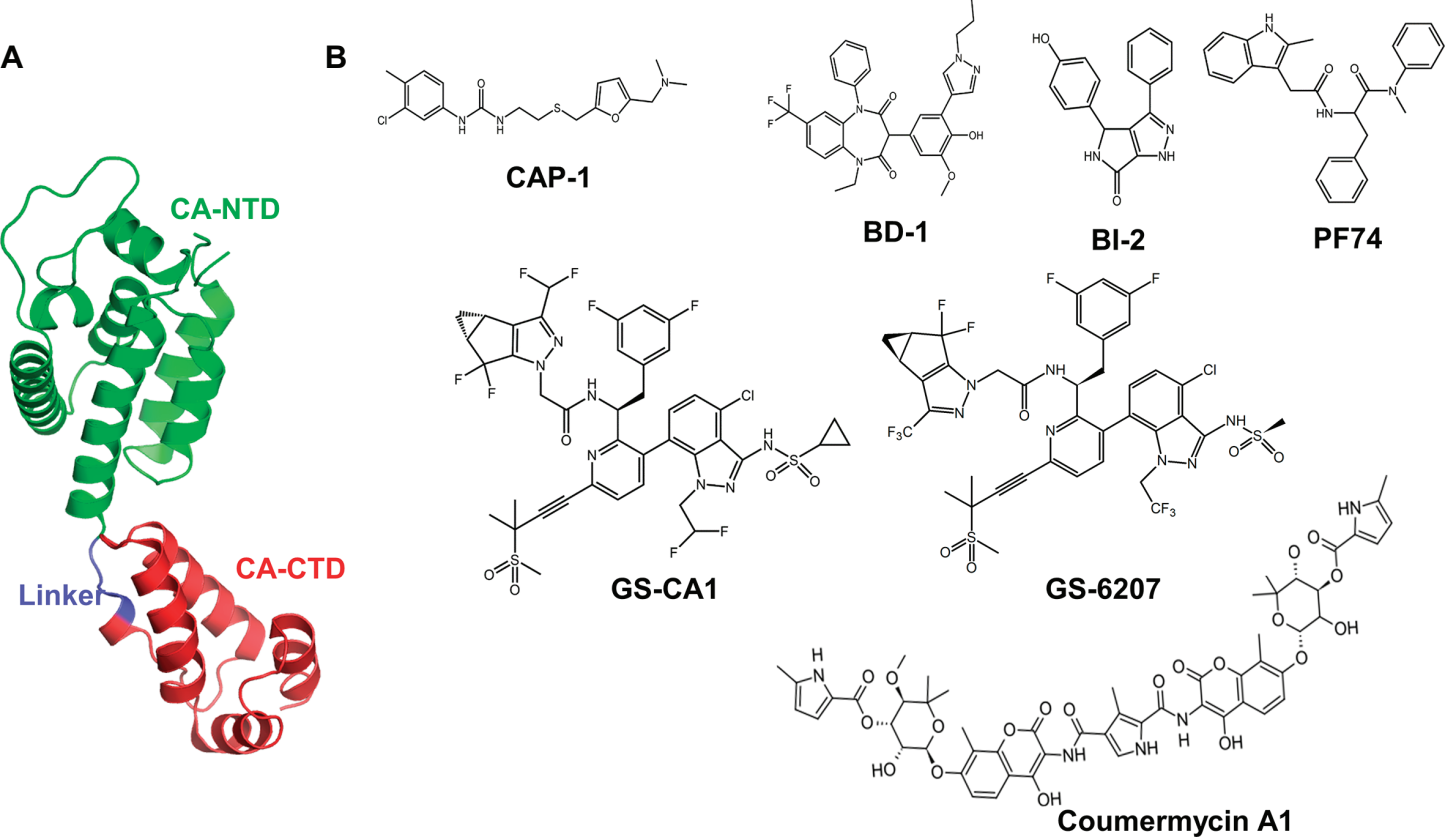
Structure of HIV-1 CA protein and representative CA inhibitors. **(A)** This figure was generated from the X-ray crystal structure of native HIV-1 capsid protein bound to PF74 (PDB entry 4XFZ) (Gres et al., 2015). NTD, N-terminal domain; CA-CTD, C-terminal domain. **(B)** Chemical structures of selective CA inhibitors. The structures of CA inhibitors shown here have either been solved in complex with CA, or we have used them in docking protocols.

HIV-1 CA has two structurally distinct domains, an N-terminal domain (CA-NTD) and a C-terminal domain (CA-CTD), which are connected by a flexible linker of ~5 residues (Figure 1A). The CA-NTD consists of seven α-helices (α1–α7), whereas CA-CTD has four α-helices (α8–α11) and a short 3_10_-helix. The structures of CA have revealed interactions between CA protomers in the form of hexameric and pentameric building blocks. The mature capsid core contains ~250 hexamers and 12 pentamers (Pornillos et al., 2009, 2011).

The capsid core is involved at multiple steps of HIV replication. Following fusion of viral and cellular membranes, the capsid core enters the cytosol, where it undergoes controlled disassembly (also known as uncoating). The timing, process, and the extent of uncoating of the capsid core are somewhat controversial. However, published reports implicate that the uncoating is associated with the initiation of reverse transcription (Campbell and Hope, 2015). CA also facilitates nuclear entry and enters the nucleus along with the preintegration complex, suggesting a role of capsid core in integration of viral DNA into the host genome (Schaller et al., 2011; Chen et al., 2016; Francis and Melikyan, 2018). During viral assembly (late stage of viral replication), the Gag polyprotein (precursor of the CA) assembles at the plasma membrane and buds as a spherical, immature, and non-infectious virus. The processing of Gag by the viral protease in several structural proteins and small peptides results in maturation of a conical capsid core.

Given the role of CA at multiple steps of viral replication, it is an attractive antiviral target, offering novel strategies for therapeutic intervention (Prevelige, 2011; Bocanegra et al., 2012; Li et al., 2013). A handful of compounds that bind HIV-1 CA have been reported (Tang et al., 2003; Sticht et al., 2005; Ternois et al., 2005; Kelly et al., 2007; Blair et al., 2010; Curreli et al., 2011; Bocanegra et al., 2012). These include benzodiazepine (BD) and benzimidazole (BM) compounds (Fader et al., 2011; Lemke et al., 2012; Tremblay et al., 2012), pyrrolopyrazolones (BI-1 and BI-2) (Lamorte et al., 2013), CAP-1 (Kelly et al., 2007; Curreli et al., 2011), and PF-3450074 (PF74) (Figure 1). The crystal structures of PF74-bound CA show that PF74 occupies a pocket between the CA-NTD and CA-CTD (Blair et al., 2010; Bhattacharya et al., 2014; Price et al., 2014). BI-2 and host factors cleavage and polyadenylation specific factor 6 (CPSF6) and nucleoporin 153 (NUP153) (Price et al., 2014) also bind in the same pocket. Therefore, both PF74 and BI-2 are expected to interfere with CA binding to CPSF6 or NUP153 (Price et al., 2012, 2014). An antibiotic Coumermycin-A1 (C-A1) has been shown to inhibit HIV-1 integration, and CA mutation A105S conferred resistance to C-A1 (Chen et al., 2016), suggesting the binding of C-A1 with CA. In addition to above-mentioned compounds, peptides (such as NYAD-1) have been reported to inhibit CA function (Tang et al., 2003; Kortagere et al., 2012; Lamorte et al., 2013; Zhang et al., 2013). Despite many promising leads, none of the compounds or peptides has advanced to clinical trials.

Recently reported CA inhibitors GS-CA1 and GS-6207 (an analog of GS-CA1, also called GS-CA2) (Figure 1) have greater potency than currently approved anti-HIV drugs (Blair et al., 2010; Tse et al., 2017; Zheng et al., 2018). GS-CA1 inhibits HIV-1 replication in T cells and peripheral blood mononuclear cells (PBMCs) at very low concentrations (EC_50_ = 240 and 140 pM, respectively). GS-6207 displays anti-HIV activity in MT-4 cells with an EC_50_ of 100 pM, whereas in PBMCs, it displays a mean EC_50_ of 50 pM (20–160 pM) against 23 HIV-1 clinical isolates from different subtypes (Zheng et al., 2018). In addition, studies in rats and dogs indicate that a single subcutaneous injection maintains GS-CA1 and GS-6207 plasma concentrations above the plasma-binding-adjusted effective concentration required for 95% HIV-1 replication inhibition for >12 weeks, indicating their potential as long-acting drugs (Jarvis, 2017; Tse et al., 2017; Carnes et al., 2018; Sager et al., 2019). Similar to PF74, GS-CA1 inhibits both early and late stages of virus replication.

The crystal structure of GS-CA1-bound CA hexamer has been reported, but it is not publicly available. Reportedly, GS-CA1 binds CA at the same general site as PF74, CPSF6, and NUP153 (Tse et al., 2017). The crystal structure of CA in complex with GS-6207 is yet to be reported. Although not well understood, GS-CA1 and GS-6207 possibly interact more extensively with CA than does PF74, providing greater binding affinity and thereby, greater efficacy than PF74. Here, using computational approaches and reported inhibitor-bound CA structures, we present the details of interactions between GS-CA compounds and CA. We find that GS-CA compounds contain structural features that are also present in PF74, BI-2, NUP153, and CPSF6. Using the same structural features in our computational modeling, we designed a cyclic peptide (Pep-1), which docked at the GS-CA binding site with comparable docking score. We validated the binding of Pep-1 to CA by determining CA binding affinity of Pep-1 using MicroScale thermophoresis (MST) experiments, which revealed that CA binds Pep-1 with ~7-fold better affinity than PF74, a well-known CA inhibitor.

## Materials and Methods

### HIV-1 CA Structure Preparation

The X-ray crystal structure of native HIV-1 capsid protein bound to PF74 (PDB entry 4XFZ) (Gres et al., 2015) was used to dock GS-CA1 and Coumermycin A1 (C-A1). Initial structures of GS-CA1 and C-A1 were generated with ChemSketch (Advanced Chemistry Development, Inc., Toronto, Ontario, Canada). These structures were subsequently minimized using MacroModel followed by LigPrep (Schrödinger Inc. NY). The PrepWizard (Schrödinger Inc. NY), which adds hydrogens, assigns bond orders, creates heteroatom states, and samples conformations of water molecules, was used to prepare CA-hexamer for docking of GS-CA1 and C-A1.

### Docking of GS-CA1, GS-6207, and C-A1

All docking simulations were conducted by the Induced-Fit Docking (IFD) module of Schrödinger Suite (Schrödinger Inc., NY). The IFD used Glide (Schrödinger Inc., NY) and the Refinement module in Prime (Schrödinger Inc., NY) to accurately predict ligand binding modes and concomitant structural changes in the receptor. A grid of 36 Å × 36 Å × 36 Å centered on the PF74 in the crystal structure of the native form of CA-hexamer (PDB file 4XFZ) for the docking of GS-CA1, GS-6207, and C-A1 was generating by the Receptor Grid Generation utility of Glide. The IFD optimized the side chain conformation to best determine the docking poses. The pose with the best IFD score was selected for comparison purposes.

### Docking of Designed Peptide Pep-1

The structure of peptide Pep-1 was generated by Prime and subjected to energy minimization using the MM/GBSA (Molecular Mechanics–Generalized Born Surface Area) method (Genheden and Ryde, 2010). The docking of the peptide into the crystal structure of CA-hexamer was conducted by IFD (Schrodinger Inc., NY). The best scoring complex of CA/Pep-1 peptide was selected for analyses. We also used PatchDock (Schneidman-Duhovny et al., 2005), though the PatchDock web server^1^ to assess if the two softwares predicted different docking conformation of Pep-1.

### MicroScale Thermophoresis Assays

The binding affinities of CA with Pep-1 and PF74 were determined by measuring thermophoresis of fluorescently labeled CA-hexamers in the presence of increasing Pep-1 or PF74 concentrations. Peptide Pep-1 was synthesized in the Molecular Interaction Core (University of Missouri) and PF74 was purchased from Sigma-Aldrich (St. Louis, MO, USA). Fluorescent labeling of CA with Alexa Fluor 647 analog NT647 was performed according to the manufacturer’s instructions (MO-L004 Monolith Protein Labeling Kit; NanoTemper Technologies GmbH, Munich, Germany). Briefly, 20 μM protein was incubated overnight with 3 M excess of dye at room temperature in a conjugation buffer provided with the labeling kit. The unreacted dye was removed by filtration through a gravity flow column provided with the kit. The elution fractions were collected in 2× MST buffer (40 mM MOPS, pH 7.2, 200 mM NaCl, and 0.2% pluronic F-127). Fluorescence intensity of each fraction was evaluated by MST (Monolith NT.115, NanoTemper Technologies GmbH, Munich, Germany), and fractions containing labeled protein were pooled. Protein concentration was determined by NanoDrop (Thermo Scientific, Waltham, MA) spectrophotometer. Aliquots were stored at −80°C until use. The reaction mixtures containing 200 nM labeled CA-hexamer and increasing concentrations of Pep-1 (1–2,000 nM) were loaded in the capillaries and the thermophoresis was monitored at 20% LED power, high MST power with 20 s MST-on time. The data were analyzed using MO. Affinity software (version 2.3) (NanoTempet Technologies, CA) by fitting the data point to a quadratic equation (Eq. 1) and plotting by Prism (Version 6.0) (GraphPad Inc., La Jolla, CA).

(1) $$F_{\mathrm{norm}}=A\;\frac{\left(K_{d}\;+\;\left[CA_{0}\right]\;+\;\left[P_{0}\right]\right)\;–\;\sqrt{\begin{array}{l} {\left(K_{d}\;+\;\left[CA_{0}\right]\;+\left[P_{0}\right]\right)}^{2}\\ –4\left[P_{0}\right]\left[CA_{0}\right] \end{array}}}{2\left[P_{0}\right]}$$

where *A* is an arbitrary parameter, *K_d_* = [*P*][*CA*]/[*P* − *CA*], [*P*] is the concentration of free Pep-1 or PF74, [*CA*] is the concentration of free CA, [*CA_0_*] is the concentration of added CA, and *P*_0_ is the concentration of added Pep-1 or PF74.

## Results and Discussion

### Interactions of GS-CA1 and GS-6207 With CA in Modeled CA/GS-CA Complexes

**Figure 2:**
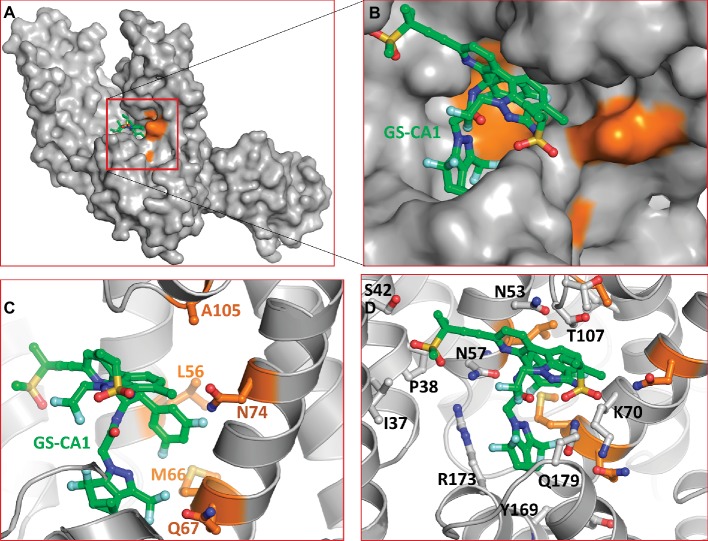
Molecular model of CA/GS-CA1 complex. **(A)** Docked pose of GS-CA1 in CA-hexamer (only dimer shown). **(B)** Close up of predicted GS-CA1 binding site in CA-hexamer. The side chains of CA and GS-CA1 are rendered as ball-and-stick. The backbone of CA is rendered as ribbons. Residues with orange carbons depict GS-CA1 resistance mutation positions (Perrier et al., 2017). **(C)** A detailed view of these residues and their proximity to GS-CA1. **(D)** Other residues of CA that interact with GS-CA1. GS-CA1 carbons in this and in subsequent figures are shown as green. The nitrogen, oxygen, sulfur, and fluorine atoms are colored blue, red, yellow, and aquamarine, respectively.

To gain insights into the interactions between the CA hexamer and GS-CA1, we used Induced-Fit Docking (IFD) interfaced with Maestro of Schrodinger Suite (Schrodinger LLC, NY) as detailed in section “Materials and Methods.” A docked pose of GS-CA1 (with best Glide score) in the crystal structure of the native form of CA-hexamer (PDB entry 4XFZ) (Gres et al., 2015) is shown in Figure 2. This figure shows that GS-CA1 binds in the close proximity to residues L56, M66, Q67, N74, and A105 (colored orange in Figures 2B,C). *In vitro* selection studies have identified GS-CA1 resistance mutations L56I, M66I, Q67H, N74D, and A105E (Perrier et al., 2017), suggesting that these mutations may affect GS-CA1 binding to the CA hexamer. Notably, IFD docking was conducted without any bias toward L56, M66, Q67, L74, or A105. In addition, in our model of the CA/GS-CA1 complex, CA-NTD residues I37, P38, S41, N53, T54, N57, Q63, L69, K70, I73, T106, T107, Y130, Y169, L172, R173, and Q179 also directly interact with GS-CA1 (Figure 2D). Many of these residues are critical to bind small molecules or peptides derived from host factors CPSF6 and NUP153 (Price et al., 2014).

In a limited size cohort (*n* = 15), the antiviral activity of GS-CA1 was reported to be comparable among clinical isolates from different subtypes (Tse et al., 2017), suggesting a strong conservation of amino acid residues in the GS-CA1 binding pocket. To assess whether the GS-CA1 binding pocket is conserved among subtypes, we generated a consensus sequence of CA from HIV-1 subtype C (HIV-1C), which accounts for more than 50% of all HIV-1 infections, using the Los Alamos HIV sequence database^2^. The results showed that the GS-CA1 binding site in HIV-1C was highly conserved. We noted only one substitution in HIV-1C (F169) compared to HIV-1B (Y169). The nearest (Cδ) atom of Y169 (or F169 in HIV-1C) is within interacting distance of GS-CA1 (< 3.8 Å), suggesting a weak interaction with GS-CA1. The effect of the change from tyrosine to phenylalanine remains to be investigated.

**Figure 3:**
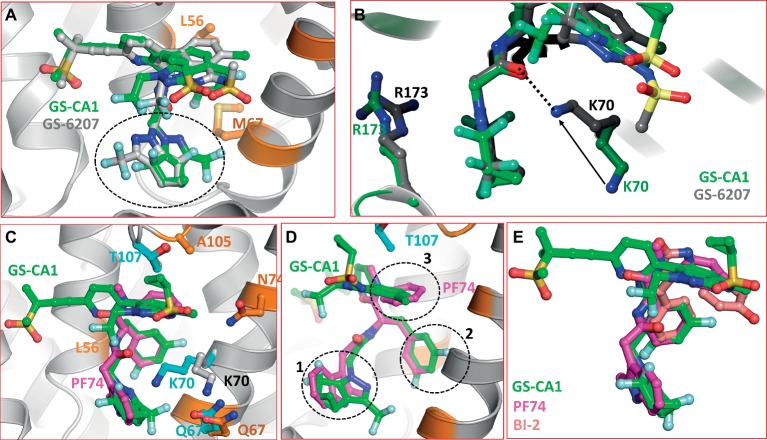
**(A)** Superposition of GS-CA1 and GS-6207. The switched position of cyclopenta-pyrazole ring is shown by dotted circle. **(B)** Difference in the side chain conformations of K70 and R173 between CA/GS-CA1 (green carbons) and CA/GS-6207 (gray carbons) complexes. Solid arrow shows the displacement of NZ atom of K73 in two complexes, whereas the dotted line shows the H-bond formed by GS-6207 with K70. This interaction is missing in CA/GS-CA1 complex. **(C)** Superposition of CA/PF74 crystal structure (PDB entry 4XZF) on the molecular model of CA/GS-CA1. The resistance mutations associated with PF74 close to the binding pocket are shown in cyan carbons. GS-CA1 resistance associated residues are depicted as in Figure 2. **(D)** Approximately 45° rotated view of Panel A. Dotted circles show superposition of three structural components of GS-CA1 and PF74 (magenta carbons). **(E)** Superposition of GS-CA1, PF74, and BI-2. The nitrogen, oxygen, sulfur, and fluorine atoms are colored blue, red, yellow, and aquamarine, respectively.

GS-6207 differs from GS-CA1 by three modifications: (1) a cyclopropane moiety on sulfonamide group was replaced by a methyl group, (2) difluoroethyl groups on indazole ring was replaced by a trifluoroethyl group, and (3) difluoromethyl group on cyclopenta-pyrazole ring was replaced by a trifluoromethyl moiety. At present, the specific rationale for these replacements is not known. We docked GS-6207 in the crystal structure of native form of CA (Gres et al., 2015). The results showed that GS-6207 binds in the same binding pocket as GS-CA1 and with a slightly better Glide score (−14.362 for GS-6207 versus −11.271 for GS-CA1), suggesting a better binding affinity. We also noted that the orientation of cyclopenta-pyrazole ring in docked GS-6207 was switched by ~180° compared to that in GS-CA1, leading to the exposure of trifluoromethyl moiety to the solvent (Figure 3A). Another remarkable difference between docked complexes of CA/GS-CA1 and CA/GS-6207 is the conformation of K70 and R173 side chains. In CA/GS-6207 complex, K70 side chain moves around 5 Å from the position in CA/GS-CA1 complex (Figure 3B, solid arrow) toward the binding pocket and forms a hydrogen bond with C=O of amide group in GS-6207 (Figure 3B, dotted line). An additional H-bond may be one of the reasons that GS-6207 has better Glide score than GS-CA1. While the side chain conformation of R173 is also altered (Figure 3B), it does not appear to be significant.

### Comparison With PF74/CA and BI-2/CA Crystal Structures

Five mutations (Q67H, K70R, T107N, L111I, and H87P) confer resistance to PF74 (Blair et al., 2010; Shi et al., 2011, 2015; Zhou et al., 2015). Residues Q67, K70, T107, and L111 reside on helices 4 and 5, whereas H87 is part of the CypA binding loop (residues 85–93) (Gamble et al., 1996; Ambrose and Aiken, 2014). The only common resistance mutation between GS-CA1 and PF74 is Q67H (Perrier et al., 2017), although other GS-CA1 resistance residues (L56, M66, L74, and A105) are also within interacting distance of PF74. A superposition of the CA/PF74 crystal structure (Gres et al., 2015) and the CA/GS-CA1 model is shown in Figure 3C. It is clear from the figure that all three rings of PF74 (two phenyl rings and one indole ring) superpose extremely well on three different rings GS-CA1 (dotted circles 1, 2, and 3 in Figure 3D). The PF74 indole ring superposes on the cyclopenta-pyrazole ring of GS-CA1 (circle 1). One of the two phenyl rings of PF74 superposes on the difluorobenzene ring of GS-CA1 (circle 2), whereas the other PF74 phenyl ring is at a topologically similar position to the indazole ring of GS-CA1 (circle 3). Additionally, the polar moieties of PF74 match topologically with the polar moieties of GS-CA1. Thus, the acetamide moiety of GS-CA1 superposes well on the corresponding moiety of PF74. These data suggest that certain structural features and interactions are common between GS-CA1 and PF74.

During IFD of GS-CA1 into the CA-hexamer, the conformations of most of the side chains in the GS-CA1/PF74 binding pocket did not change significantly as compared to the CA/PF74 crystal structure, with the exception of the side chain of K70 (Figure 3C). The position of the K70 NZ atom was shifted by ~4.7 Å from its position in the CA/PF74 complex (cyan versus gray carbons in Figure 3C), suggesting an absence of interactions between K70 and GS-CA1, in contrast to K70 interactions with PF74. The absence of this interaction is a possible reason that mutation at K70 did not emerge during GS-CA1 *in vitro* resistance selection studies (Perrier et al., 2017). As mentioned above, the interaction of K70 is restored in the CA/GS-6207 complex. At present, the resistance mutation profile of GS-6207 is not known. Hence, the significance of this interaction awaits virological studies.

BI-2 is one of the two 4, 5-dihydro-1H-pyrrolo [3,4-c]pyrazol-6-one series compounds shown to bind the CA hexamer. BI-2 was shown to stabilize CA hexamers and inhibit HIV-1 at early stages of infection (Lamorte et al., 2013). Selection of viruses resistant to BI-2 identified mutations at residues A105 and T107 of CA-NTD (Lamorte et al., 2013). The high resolution structure of CA in complex with BI-2 showed that it binds at the PF74 binding site. The superposition of the three compounds (GS-CA1, PF74, and BI-2) obtained from the superposition of Cα-atoms of CA-NTD showed that the three compounds have a common binding mode with CA-hexamer (Figure 3E). Our docking results of GS-CA1 showed that CA residue A105 is within interacting distance of GS-CA1, and the common resistance mutation A105T between GS-CA1 and BI-2 further confirms that the two compounds share part of the binding site.

### Comparison With CPSF6/CA and NUP153/CA Crystal Structures

**Figure 4:**
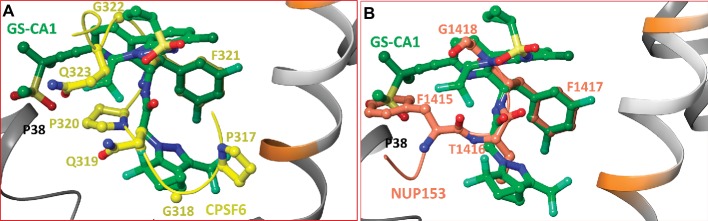
**(A)** Superposition of CA/CPSF6 crystal structure on the molecular model of CA/GS-CA1. For clarity, residues P313-P316 and backbone atoms of CPSF6 have been omitted. Peptide CPSF6 is shown in yellow carbons, and the backbone of CPSF6 peptide is shown as a yellow tube. This figure shows the superposition of the difluorobenzyl ring of GS-CA1 on F321 of CPSF6. **(B)** Superposition of CA/NUP153 crystal structure complex on the molecular model of CA/GS-CA1. For clarity, only residues F1415-G1418 are shown. The carbon atoms and backbone (tube) of NUP153 are shown in faded red-orange color. This figure shows the superposition of the difluorobenzyl ring of GS-CA1 on F1417 of NUP153. In addition, F1415 of NUP153 and the methylsulfonyl group of GS-CA1 have a common interaction with P38 of CA-hexamer. The atoms of P38 are not shown for clarity.

The crystal structures of CA in complex with short peptides derived from CPSF6 and NUP153 showed that both peptides share the binding pocket occupied by PF74 and BI-2 (Price et al., 2014), although the bound peptides had additional interactions. To determine whether common structural features among GS-CA1, CPSF6, and NUP153 exist upon binding to CA, we superimposed the crystal structures of CA/CPSF6 and CA/NUP153 on our modeled CA/GS-CA1 complex. The superposition is shown in Figure 4, demonstrating that the conformation of GS-CA1 docked into the CA-hexamer follows the folding of the CPSF6 peptide (Figure 4A). Remarkably, the side chain of F321 of CPSF6 perfectly superposed on the difluorobenzyl moiety of GS-CA1.

Similar to CPSF6, the NUP153 backbone follows the conformation of GS-CA1, and F1417 of NUP153 perfectly superposes on the difluorobenzyl moiety of GS-CA1 (Figure 4B). In addition, there exists a hydrophobic interaction between the methylsulfonyl moiety of GS-CA1 and P38 of CA (atoms of P38 are not shown). A similar interaction is noted between F1415 of NUP153 and P38 (Figure 4B).

### Comparison With CA/CAP-1, CA/BD, and CA/BM Complexes

**Figure 5:**
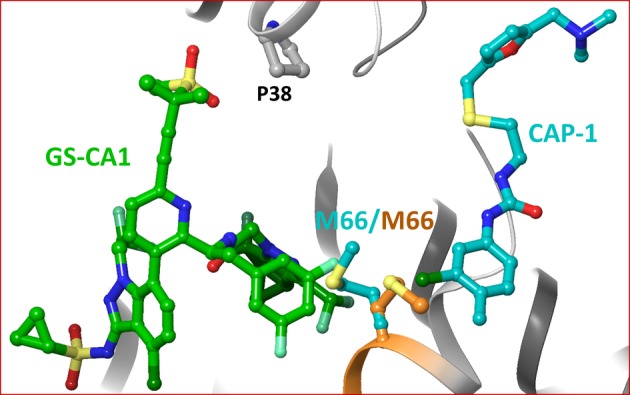
Superposition of CA/CAP-1 crystal structure complex on the molecular model of CA/GS-CA1. For clarity, only M66 in the two structures is shown (orange – CA/GS-CA1; teal – CA/CAP-1).

CAP-1 1-(3-chloro-4-methylphenyl)-3-(2-(((5-((dimethylamino)methyl)furan-2-yl)methyl)thio)ethyl)urea is an assembly inhibitor for which the resistance mutation profile has not been reported (Kelly et al., 2007). The structure of CAP-1 bound CA-NTD has been solved by NMR and X-ray crystallography (Kelly et al., 2007). A comparison of the crystal and NMR structures demonstrated that CA undergoes significant conformational change upon CAP-1 binding. The superposition of the crystal structure of the CA/CAP-1 complex on the model structure of the CA/GS-CA1 complex showed that the two inhibitors did not bind at a common site (Figure 5). However, two residues (M66 and L69) interacted with both GS-CA1 and CAP-1. The positions of M66 in the CA/GS-CA1 and CA/CAP-1 complexes are shown in Figure 5. The compounds of the benzodiazepine (BD1–BD4) and benzimidazole (BM1–BM5) series bind to CA at a site that is close to the CAP-1 binding site (Lemke et al., 2012). While compounds from both series have been shown to bind at the same pocket, they have distinct resistance mutation profiles. Mutations V36T and G61E were selected with BD inhibitors, whereas K30R and S33G were selected with BM inhibitors. Both V36 and G61 are part of BM3 binding pocket (PDB entry 4E91) (Sticht et al., 2005). K30 is not within the interacting distance of BM4, and the backbone carbonyl group of S33 only forms a Van der Waals interaction with BM4 (PDB entry 4E92) (Lemke et al., 2012). Hence, the resistance mechanism of BM4 does not seem to operate through direct interactions. The crystal structures of BD3 and BM4 bound to CA-NTD showed that both compounds are within interacting distance of M66, similar to CAP-1. Hence, BD and BM series compounds do not share a binding site with GS-CA1, but they all have a common interaction with M66.

### Coumermycin A1 Binding to CA-Hexamers

Coumermycin A1 (C-A1) is a gyrase B inhibitor that also inhibits HSP90 (Vozzolo et al., 2010) (reviewed in Carnes et al., 2018). A crystal structure of CA/C-A1 has not been solved. However, docking studies predict the binding of C-A1 in a pocket formed by two adjacent capsid monomers (Chen et al., 2016). This predicted binding site may be relevant, as mutations N74D and A105S conferred resistance to C-A1, and both residues (N74 and A105) are at the interface of two capsid monomers.

We used IFD to assess the details of interactions between C-A1 and CA. Of 32 predicted docking poses of C-A1 in the same PDB file (4XFZ) as used by Chen et al. (2016), none of the poses were within interacting distance of N74 or A105. Our docking data predict that that resistance of N74D and A105S to C-A1 may not be due to binding defects imparted by mutations at these residues.

### Other Small Molecule Inhibitors of CA and Their Comparison With the Binding of GS-CA1

Several additional CA inhibitors have been reported, such as CK026, I-XW-053, compound 34 (Kortagere et al., 2012), C1 (Lemke et al., 2013), and Ebselen (Lemke et al., 2013). CK026 is a large molecule, and was not shown to inhibit HIV-1 in PBMCs. However, I-XW-053 and compound 34, derivatives of CK026, demonstrated inhibitory activities in PBMCs (Kortagere et al., 2014). A crystal structure of CA in complex with these compounds has not been solved. However, the docking results in combination with binding affinity determination *via* surface plasmon resonance revealed that compound 34 binds in the vicinity of P38, S41, R173, K170, and Q179 (Kortagere et al., 2014). All of these residues are within interacting distance of GS-CA1 in our modeled CA/GS-CA1 complex (Figure 2).

Compound C1 has been shown to bind at a unique site near the CypA-binding loop and affects late steps by disrupting proper assembly of mature capsid (Lemke et al., 2013). However, the crystal structures of CA in the presence of compound C1 and BD series compounds show that C1 induces CA dimer formation and binds at the interface of the dimer. Mutation R132T confers resistance to C1. In the crystal structures of C1 and BD/BM compounds, R132 forms a polar interaction with compound C1. These structures also show that C1 makes contact with the N-terminus of helix 2, forming hydrophobic interactions with P34, G35, I37, and P38. The benzoic acid moiety forms a direct hydrogen bond to A139, and there is a water-mediated hydrogen bond to S41 (Lemke et al., 2013). Both I37 and P38 form hydrophobic interactions with GS-CA1 (Figure 2).

Ebselen is a small molecule that was discovered in a search for inhibitors of CA dimerization. Electrospray ionization mass spectrometry experiments revealed that ebselen covalently binds CA-CTD, most likely through a selenylsulfide linkage involving C198 and C218 (Thenin-Houssier et al., 2016). Both of these residues are part of the CA-CTD, and they are not within interacting distance of the GS-CA1 in our modeled CA/GS-CA1 complex. Therefore, we predict that ebselen and GS-CA1 binding sites do not overlap.

### Docking of a Designed Cyclic Peptide Inhibitor (Pep-1)

Using the crystal structures of PF74, NUP153, CPSF6, and BI-2-bound CA as well as the modeled structure of the CA/GS-CA1 (CA/GS-6207) complex, we designed a cyclic peptide, Pep-1, containing common structural components/groups among CA-bound small molecules or peptides derived from CPSF6 and NUP153. The docking of Pep-1 showed that it binds in a pocket that is shared by PF74, NUP153, CPSF6, GS-CA1, and GS-6207. The structural components that superposed in different complexes are listed in Table 1. It appears that the designed peptide shares binding site and chemical moieties that may inhibit CA function. The structural and chemical details of Pep-1 will be reported elsewhere. However, we determined the binding affinity of CA with Pep-1 and compared with the PF74 binding affinity (presented below).

**Table 1:** Common structural groups/components in different CA complexes.

PF74	BI-2	CPSF6	NUP153	GS-CA1	Pep-1
Phenyl	Phenyl	F321	F1417	Difluorobenzyl	Phenylalanine
Phenyl	Phenol	–	–	Indazole	Proline
Indole	–	G318-Q319^1^	–	Cyclopenta-pyrazole	Valine
–	–		F1415	Methylsulfonyl	Phenylalanine

1 *A part of G318-Q319 is topologically close to the indole ring of PF74*.

### Binding Affinity of CA With Pep-1 and PF74

**Figure 6:**
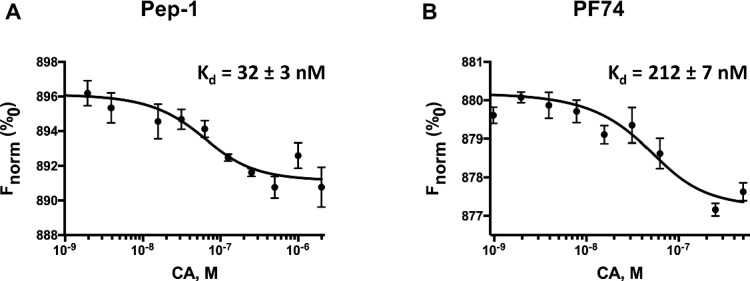
Pep-1 **(A)** and PF74 **(B)** binding affinities of CA as determined by MicroScale thermophoresis. This figure shows the change in fluorescence due to thermophoresis at the increasing concentrations of Pep-1 and PF74 (1–2,000 nM) in the presence of 200 nM CA-hexamers.

We used the MicroScale thermophoresis (MST) assays to determine the binding affinity of CA to Pep-1 and PF74. MST is based on the thermophoresis, a directed movement of molecules in a temperature gradient, which depends on a variety of molecular properties including size, charge, hydration shell, and conformation. Thus, it is highly sensitive to virtually any change in molecular properties, allowing for precise quantification of molecular events independent of the size or nature of the investigated sample (Jerabek-Willemsen et al., 2014). During the MST experiment, a temperature gradient is induced by an infrared laser. The directed movement of molecules through the temperature gradient is detected and quantified using a covalently attached fluorophore. The binding isotherms obtained by plotting the difference in normalized fluorescence against increasing Pep-1 and PF74 concentration are shown in Figures 6A,B, respectively. The binding affinities of Pep-1 (K_d.Pep-1_) and PF74 (K_d.PF74_) with CA were extrapolated by fitting the data points to a quadratic equation (Eq. 1). The *K*_*d*.Pep-1_ from these data is 32 ± 3 nM, whereas the *K*_*d*.PF74_ is 212 ± 7 nM. The binding affinity of CA-hexamers with PF74 was previously determined by isothermal calorimetry (ITC) to be 262 nM (Bhattacharya et al., 2014), which is in good agreement with the *K*_*d*.PF74_ determined here using MST. These data suggest that Pep-1 binds CA with ~7-fold greater affinity.

## Conclusion

The results obtained from flexible docking and comparative structural analyses presented above show that the compounds GS-CA1 and GS-6207 contain structural features that are present in several previously discovered small molecule inhibitors of CA, as well as in CA-interacting host factors. Most importantly, the phenyl moieties of PF74 and BI-2 perfectly superpose on the difluorobenzyl group of GS-CA1 and GS-6207, as do the phenylalanine residues of CPSF6 and NUP153 peptides. The position of the phenyl ring is critical in binding of these compounds, as it forms direct contacts with critical CA residues L56, N57, and M66. A significantly larger size of GS-CA1 and GS-6207 compared to PF74 likely ensures additional contacts with CA. These contacts are either present in CA/CPSF6, CA/NUP153, CA/BD (or CA/BM), or CA/CAP-1 crystal structures. Collectively, it appears that GS-CA compounds have been designed to include structural features of many CA-binding molecules to ensure that they mimic the interactions of many ligands. Furthermore, we suggest that a cyclic peptide designed based on the structures of small molecules inhibitors and host factors bound CA has strong binding affinity to CA.

## Data Availability

The raw data supporting the conclusions of this manuscript will be made available by the authors, without undue reservation, to any qualified researcher.

## Author Contributions

KS and AS conceived and designed the study. UN conducted sequence analyses, and KS conducted computational analyses. KS, FG, TQ, and UN analyzed the results. KS wrote the original manuscript. KS, KH, ML, TQ, DB, UN, and AS edited the manuscript.

### Conflict of Interest Statement

The authors declare that the research was conducted in the absence of any commercial or financial relationships that could be construed as a potential conflict of interest.

## Footnotes

^1^https://bioinfo3d.cs.tau.ac.il/PatchDock/

^2^https://www.hiv.lanl.gov

## References

[ref1] ACD/ChemSketch (Freeware), version 2017.1.2. (2017). Toronto, ON, Canada: Advanced Chemistry Devlopment, Inc. Available at: www.acdlabs.com (Accessed June 22, 2017).

[ref2] Ambrose Z. Aiken C. (2014). HIV-1 uncoating: connection to nuclear entry and regulation by host proteins. Virology 454–455, 371–379. 10.1016/j.virol.2014.02.004, PMID: 24559861 PMC3988234

[ref3] Antiretroviral Therapy Cohort Collaboration (2017). Survival of HIV-positive patients starting antiretroviral therapy between 1996 and 2013: a collaborative analysis of cohort studies. Lancet HIV 4, e349–e356. 10.1016/S2352-3018(17)30066-8 28501495 PMC5555438

[ref4] Bhattacharya A. Alam S. L. Fricke T. Zadrozny K. Sedzicki J. Taylor A. B.. (2014). Structural basis of HIV-1 capsid recognition by PF74 and CPSF6. Proc. Natl. Acad. Sci. USA 111, 18625–18630. 10.1073/pnas.1419945112, PMID: 25518861 PMC4284599

[ref5] Blair W. S. Pickford C. Irving S. L. Brown D. G. Anderson M. Bazin R.. (2010). HIV capsid is a tractable target for small molecule therapeutic intervention. PLoS Pathog. 6:e1001220. 10.1371/journal.ppat.1001220, PMID: 21170360 PMC3000358

[ref6] Bocanegra R. Rodriguez-Huete A. Fuertes M. A. Del Alamo M. Mateu M. G. (2012). Molecular recognition in the human immunodeficiency virus capsid and antiviral design. Virus Res. 169, 388–410. 10.1016/j.virusres.2012.06.016, PMID: 22728445

[ref7] Campbell E. M. Hope T. J. (2015). HIV-1 capsid: the multifaceted key player in HIV-1 infection. Nat. Rev. Microbiol. 13, 471–483. 10.1038/nrmicro3503, PMID: 26179359 PMC4876022

[ref8] Carnes S. K. Sheehan J. H. Aiken C. (2018). Inhibitors of the HIV-1 capsid, a target of opportunity. Curr. Opin. HIV AIDS 13, 359–365. 10.1097/COH.0000000000000472, PMID: 29782334 PMC6075716

[ref9] Chen N. Y. Zhou L. Gane P. J. Opp S. Ball N. J. Nicastro G. (2016). HIV-1 capsid is involved in post-nuclear entry steps. Retrovirology 13:28. 10.1186/s12977-016-0262-0 27107820 PMC4842275

[ref10] Curreli F. Zhang H. Zhang X. Pyatkin I. Victor Z. Altieri A.. (2011). Virtual screening based identification of novel small-molecule inhibitors targeted to the HIV-1 capsid. Bioorg. Med. Chem. 19, 77–90. 10.1016/j.bmc.2010.11.045, PMID: 21168336 PMC3034313

[ref11] Fader L. D. Bethell R. Bonneau P. Bos M. Bousquet Y. Cordingley M. G.. (2011). Discovery of a 1,5-dihydrobenzo[b][1,4]diazepine-2,4-dione series of inhibitors of HIV-1 capsid assembly. Bioorg. Med. Chem. Lett. 21, 398–404. 10.1016/j.bmcl.2010.10.131, PMID: 21087861

[ref12] Francis A. C. Melikyan G. B. (2018). Single HIV-1 imaging reveals progression of infection through CA-dependent steps of docking at the nuclear pore, uncoating, and nuclear transport. Cell Host Microbe 23, 536–548. e536. 10.1016/j.chom.2018.03.009, PMID: 29649444 PMC5901770

[ref13] Gamble T. R. Vajdos F. F. Yoo S. Worthylake D. K. Houseweart M. Sundquist W. I.. (1996). Crystal structure of human cyclophilin A bound to the amino-terminal domain of HIV-1 capsid. Cell 87, 1285–1294. 10.1016/S0092-8674(00)81823-1, PMID: 8980234

[ref14] Genheden S. Ryde U. (2010). How to obtain statistically converged MM/GBSA results. J. Comput. Chem. 31, 837–846. 10.1002/jcc.21366, PMID: 19598265

[ref15] Gres A. T. Kirby K. A. Kewalramani V. N. Tanner J. J. Pornillos O. Sarafianos S. G. (2015). X-ray crystal structures of native HIV-1 capsid protein reveal conformational variability. Science 349, 99–103. 10.1126/science.aaa5936, PMID: 26044298 PMC4584149

[ref16] Harries A. D. Suthar A. B. Takarinda K. C. Tweya H. Kyaw N. T. Tayler-Smith K. (2016). Ending the HIV/AIDS epidemic in low- and middle-income countries by 2030: is it possible? F1000Res 5:2328. 10.12688/f1000research.9247.1 27703672 PMC5031124

[ref17] Jarvis L. M. (2017). Conquering HIV's capsid. Chem. Eng. News 95, 23–25.

[ref18] Jerabek-Willemsen M. T. Wanner R. Roth H. M. Duhr S. Baaske P. Breitsprecher D. (2014). MicroScale thermophoresis: interaction analysis and beyond. J. Mol. Struct. 1077, 101–113. 10.1016/j.molstruc.2014.03.009

[ref19] Kelly B. N. Kyere S. Kinde I. Tang C. Howard B. R. Robinson H.. (2007). Structure of the antiviral assembly inhibitor CAP-1 complex with the HIV-1 CA protein. J. Mol. Biol. 373, 355–366. 10.1016/j.jmb.2007.07.070, PMID: 17826792 PMC2066180

[ref20] Kortagere S. Madani N. Mankowski M. K. Schon A. Zentner I. Swaminathan G.. (2012). Inhibiting early-stage events in HIV-1 replication by small-molecule targeting of the HIV-1 capsid. J. Virol. 86, 8472–8481. 10.1128/JVI.05006-11, PMID: 22647699 PMC3421734

[ref21] Kortagere S. Xu J. P. Mankowski M. K. Ptak R. G. Cocklin S. (2014). Structure-activity relationships of a novel capsid targeted inhibitor of HIV-1 replication. J. Chem. Inf. Model. 54, 3080–3090. 10.1021/ci500437r, PMID: 25302989 PMC4245176

[ref22] Lamorte L. Titolo S. Lemke C. T. Goudreau N. Mercier J. F. Wardrop E.. (2013). Discovery of novel small-molecule HIV-1 replication inhibitors that stabilize capsid complexes. Antimicrob. Agents Chemother. 57, 4622–4631. 10.1128/AAC.00985-13, PMID: 23817385 PMC3811413

[ref23] Lemke C. T. Titolo S. Goudreau N. Faucher A. M. Mason S. W. Bonneau P. (2013). A novel inhibitor-binding site on the HIV-1 capsid N-terminal domain leads to improved crystallization via compound-mediated dimerization. Acta Crystallogr. D Biol. Crystallogr. 69, 1115–1123. 10.1107/S0907444913006409, PMID: 23695256

[ref24] Lemke C. T. Titolo S. Von Schwedler U. Goudreau N. Mercier J. F. Wardrop E.. (2012). Distinct effects of two HIV-1 capsid assembly inhibitor families that bind the same site within the N-terminal domain of the viral CA protein. J. Virol. 86, 6643–6655. 10.1128/JVI.00493-12, PMID: 22496222 PMC3393593

[ref25] Li G. Verheyen J. Rhee S. Y. Voet A. Vandamme A. M. Theys K. (2013). Functional conservation of HIV-1 Gag: implications for rational drug design. Retrovirology 10:126. 10.1186/1742-4690-10-126, PMID: 24176092 PMC4228425

[ref26] May M. T. Gompels M. Delpech V. Porter K. Orkin C. Kegg S.. (2014). Impact on life expectancy of HIV-1 positive individuals of CD4+ cell count and viral load response to antiretroviral therapy. AIDS 28, 1193–1202. 10.1097/QAD.0000000000000243, PMID: 24556869 PMC4004637

[ref27] Perrier M. Bertine M. Le Hingrat Q. Joly V. Visseaux B. Collin G.. (2017). Prevalence of gag mutations associated with in vitro resistance to capsid inhibitor GS-CA1 in HIV-1 antiretroviral-naive patients. J. Antimicrob. Chemother. 72, 2954–2955. 10.1093/jac/dkx208, PMID: 29091184

[ref28] Pornillos O. Ganser-Pornillos B. K. Kelly B. N. Hua Y. Whitby F. G. Stout C. D. (2009). X-ray structures of the hexameric building block of the HIV capsid. Cell 137, 1282–1292. 10.1016/j.cell.2009.04.063 19523676 PMC2840706

[ref29] Pornillos O. Ganser-Pornillos B. K. Yeager M. (2011). Atomic-level modelling of the HIV capsid. Nature 469, 424–427. 10.1038/nature09640, PMID: 21248851 PMC3075868

[ref30] Prevelige P. E. Jr. (2011). New approaches for antiviral targeting of HIV assembly. J. Mol. Biol. 410, 634–640. 10.1016/j.jmb.2011.03.074, PMID: 21762804 PMC3139149

[ref31] Price A. J. Fletcher A. J. Schaller T. Elliott T. Lee K. Kewalramani V. N.. (2012). CPSF6 defines a conserved capsid interface that modulates HIV-1 replication. PLoS Pathog. 8:e1002896. 10.1371/journal.ppat.1002896, PMID: 22956906 PMC3431306

[ref32] Price A. J. Jacques D. A. Mcewan W. A. Fletcher A. J. Essig S. Chin J. W.. (2014). Host cofactors and pharmacologic ligands share an essential interface in HIV-1 capsid that is lost upon disassembly. PLoS Pathog. 10:e1004459. 10.1371/journal.ppat.1004459, PMID: 25356722 PMC4214760

[ref33] Quinn T. C. (2008). HIV epidemiology and the effects of antiviral therapy on long-term consequences. AIDS 22(Suppl. 3), S7–S12. 10.1097/01.aids.0000327510.68503.e8, PMID: 18845925 PMC2753265

[ref34] Sabin C. A. (2013). Do people with HIV infection have a normal life expectancy in the era of combination antiretroviral therapy? BMC Med. 11:251. 10.1186/1741-7015-11-251 24283830 PMC4220799

[ref35] Sager J. E. Begley R. Rhee M. West S. K. Ling J. Schroeder S. D. (2019). “Safety and pK of subcutaneous GS-6207, a novel HIV-1 capsid inhibitor (Abstract #141)” in Conference on Retroviruses and Opportunistic Infections, March 4–7, Seattle, WA, USA.

[ref36] Schaller T. Ocwieja K. E. Rasaiyaah J. Price A. J. Brady T. L. Roth S. L.. (2011). HIV-1 capsid-cyclophilin interactions determine nuclear import pathway, integration targeting and replication efficiency. PLoS Pathog. 7:e1002439. 10.1371/journal.ppat.1002439, PMID: 22174692 PMC3234246

[ref37] Schneidman-Duhovny D. Inbar Y. Nussinov R. Wolfson H. J. (2005). PatchDock and SymmDock: servers for rigid and symmetric docking. Nucleic Acids Res. 33, W363–W367. 10.1093/nar/gki481, PMID: 15980490 PMC1160241

[ref38] Shi J. Zhou J. Halambage U. D. Shah V. B. Burse M. J. Wu H. (2015). Compensatory substitutions in the HIV-1 capsid reduce the fitness cost associated with resistance to a capsid-targeting small-molecule inhibitor. J. Virol. 89, 208–219. 10.1128/JVI.01411-14 25320302 PMC4301104

[ref39] Shi J. Zhou J. Shah V. B. Aiken C. Whitby K. (2011). Small-molecule inhibition of human immunodeficiency virus type 1 infection by virus capsid destabilization. J. Virol. 85, 542–549. 10.1128/JVI.01406-10 20962083 PMC3014163

[ref40] Sticht J. Humbert M. Findlow S. Bodem J. Muller B. Dietrich U.. (2005). A peptide inhibitor of HIV-1 assembly in vitro. Nat. Struct. Mol. Biol. 12, 671–677. 10.1038/nsmb964, PMID: 16041387

[ref41] Tang C. Loeliger E. Kinde I. Kyere S. Mayo K. Barklis E.. (2003). Antiviral inhibition of the HIV-1 capsid protein. J. Mol. Biol. 327, 1013–1020. 10.1016/S0022-2836(03)00289-4, PMID: 12662926

[ref42] Teeraananchai S. Kerr S. J. Amin J. Ruxrungtham K. Law M. G. (2017). Life expectancy of HIV-positive people after starting combination antiretroviral therapy: a meta-analysis. HIV Med. 18, 256–266. 10.1111/hiv.12421, PMID: 27578404

[ref43] Ternois F. Sticht J. Duquerroy S. Krausslich H. G. Rey F. A. (2005). The HIV-1 capsid protein C-terminal domain in complex with a virus assembly inhibitor. Nat. Struct. Mol. Biol. 12, 678–682. 10.1038/nsmb967, PMID: 16041386

[ref44] Thenin-Houssier S. De Vera I. M. Pedro-Rosa L. Brady A. Richard A. Konnick B.. (2016). Ebselen, a small-molecule capsid inhibitor of HIV-1 replication. Antimicrob. Agents Chemother. 60, 2195–2208. 10.1128/AAC.02574-15, PMID: 26810656 PMC4808204

[ref45] Tremblay M. Bonneau P. Bousquet Y. Deroy P. Duan J. Duplessis M.. (2012). Inhibition of HIV-1 capsid assembly: optimization of the antiviral potency by site selective modifications at N1, C2 and C16 of a 5-(5-furan-2-yl-pyrazol-1-yl)-1H-benzimidazole scaffold. Bioorg. Med. Chem. Lett. 22, 7512–7517. 10.1016/j.bmcl.2012.10.034, PMID: 23122820

[ref46] Tse W. C. Link J. O. Mulato A. Niedziela-Majka A. Rowe W. Somoza J. R. (2017). “Discovery of novel potent HIV capsid inhibitors with long-acting potential” in Conference on Retroviruses and Opportunistic Infections Abstract No. 38, Feb. 13–16, 2017 (Washington: Seattle).

[ref47] Vozzolo L. Loh B. Gane P. J. Tribak M. Zhou L. Anderson I.. (2010). Gyrase B inhibitor impairs HIV-1 replication by targeting Hsp90 and the capsid protein. J. Biol. Chem. 285, 39314–39328. 10.1074/jbc.M110.155275, PMID: 20937817 PMC2998086

[ref48] Zhang H. Curreli F. Waheed A. A. Mercredi P. Y. Mehta M. Bhargava P.. (2013). Dual-acting stapled peptides target both HIV-1 entry and assembly. Retrovirology 10:136. 10.1186/1742-4690-10-136, PMID: 24237936 PMC3842668

[ref49] Zheng J. Yant S. R. Ahmadyar S. Chan T. Y. Chiu A. Cihlar T. (2018). 539. GS-CA2: a novel, potent, and selective first-in-class inhibitor of HIV-1 capsid function displays nonclinical pharmacokinetics supporting long-acting potential in humans. Open Forum Infect. Dis. 5, S199–S200. 10.1093/ofid/ofy210.548

[ref50] Zhou J. Price A. J. Halambage U. D. James L. C. Aiken C. (2015). HIV-1 resistance to the capsid-targeting inhibitor PF74 results in altered dependence on host factors required for virus nuclear entry. J. Virol. 89, 9068–9079. 10.1128/JVI.00340-15, PMID: 26109731 PMC4524096

